# Analysis of chromosomal structural variations in patients with recurrent spontaneous abortion using optical genome mapping

**DOI:** 10.3389/fgene.2023.1248755

**Published:** 2023-09-04

**Authors:** Huihua Rao, Haoyi Zhang, Yongyi Zou, Pengpeng Ma, Tingting Huang, Huizhen Yuan, Jihui Zhou, Wan Lu, Qiao Li, Shuhui Huang, Yanqiu Liu, Bicheng Yang

**Affiliations:** ^1^ Department of Medical Genetics, Jiangxi Maternal and Child Health Hospital, Nanchang, Jiangxi, China; ^2^ Jiangxi Key Laboratory of Birth Defect Prevention and Control, Jiangxi Maternal and Child Health Hospital, Nanchang, Jiangxi, China; ^3^ School of Public Health, Nanchang University, Nanchang, Jiangxi, China

**Keywords:** recurrent spontaneous abortion (RSA), structural variations (SVs), optical genome mapping (OGM), Oxford Nanopore technology (ONT), balanced reciprocal translocation (BRT), complex chromosomal rearrangement (CCR)

## Abstract

**Background and aims:** Certain chromosomal structural variations (SVs) in biological parents can lead to recurrent spontaneous abortions (RSAs). Unequal crossing over during meiosis can result in the unbalanced rearrangement of gamete chromosomes such as duplication or deletion. Unfortunately, routine techniques such as karyotyping, fluorescence *in situ* hybridization (FISH), chromosomal microarray analysis (CMA), and copy number variation sequencing (CNV-seq) cannot detect all types of SVs. In this study, we show that optical genome mapping (OGM) quickly and accurately detects SVs for RSA patients with a high resolution and provides more information about the breakpoint regions at gene level.

**Methods:** Seven couples who had suffered RSA with unbalanced chromosomal rearrangements of aborted embryos were recruited, and ultra-high molecular weight (UHMW) DNA was isolated from their peripheral blood. The consensus genome map was created by *de novo* assembly on the Bionano Solve data analysis software. SVs and breakpoints were identified via alignments of the reference genome GRCh38/hg38. The exact breakpoint sequences were verified using either Oxford Nanopore sequencing or Sanger sequencing.

**Results:** Various SVs in the recruited couples were successfully detected by OGM. Also, additional complex chromosomal rearrangement (CCRs) and four cryptic balanced reciprocal translocations (BRTs) were revealed, further refining the underlying genetic causes of RSA. Two of the disrupted genes identified in this study, *FOXK2* [46,XY,t(7; 17)(q31.3; q25)] and *PLXDC2* [46,XX,t(10; 16)(p12.31; q23.1)], had been previously shown to be associated with male fertility and embryo transit.

**Conclusion:** OGM accurately detects chromosomal SVs, especially cryptic BRTs and CCRs. It is a useful complement to routine human genetic diagnostics, such as karyotyping, and detects cryptic BRTs and CCRs more accurately than routine genetic diagnostics.

## Introduction

Recurrent spontaneous abortion (RSA) is defined as the loss of two or more consecutive pregnancies. RSA affects 1%–5% of child-bearing age women (Green and Donoghue, 2019). The complex etiology of RSA is related to genetics, immune and endocrine systems, anatomical structure of reproductive organs, environment, and other factors (Stephenson, 1996). Parental chromosomal structural variations (SVs) are the major genetic cause of early spontaneous abortion (Ozawa et al., 2019). Typically, carriers are phenotypically normal but have a significantly increased risk of miscarriage. This is caused by unbalanced rearrangements of the gamete chromosomes, such as duplications or deletions resulting from unequal crossing over during meiosis (Soltani et al., 2021). In addition, certain breakpoints of SVs may disrupt haploinsufficient genes or their regulatory elements, placing the carriers at risk for neurodevelopmental or other clinical diseases (Schluth-Bolard et al., 2013; Northcott et al., 2014; Nilsson et al., 2017; Northcott et al., 2017).

Regular cytogenetic testing has been used as a confirmatory diagnostic to detect apparent chromosomal rearrangements. However, G-banding karyotyping is time-consuming, and its resolution is limited to >5–10 Mb, rendering cryptic SVs hard to identify (Dremsek et al., 2021). Previous studies have found that 2%–4% of RSA couples carry chromosomal abnormalities (Fryns and Van Buggenhout, 1998; Popescu et al., 2018), but the actual number of chromosomal variants undetectable by routine karyotyping is much higher than that (Dong et al., 2019). Compared with the low resolution of karyotyping, genome copy number variation sequencing (CNV-seq) and chromosomal microarray analysis (CMA) can detect copy number variations (CNVs) at submicroscopic level, which improves the detection of microdeletions/microduplications in abortion tissues (Bajaj Lall et al., 2021). However, CNV-seq/CMA cannot detect balanced chromosomal abnormalities. While they can be used to indirectly determine whether a parent carries a chromosomal rearrangement, the results for embryos’ CNVs are often inconclusive. Fluorescence *in situ* hybridization (FISH) is a feasible method to verify the structural variations, but its application is limited by its inability to detect unknown SVs and the difficulty of obtaining specific fluorescent probes (Cui et al., 2016). Currently, whole-genome sequencing (WGS) enables detection of SVs. However, the detection rate is imperfect and limited by the short-read length and the repetitive nature of sequences at some SV breakpoints, since many of them are mediated by non-allelic homologous recombination of repeats (Kosugi et al., 2019; Savara et al., 2021).

Optical genome mapping (OGM) is a new technology that uses ultra-long linear single DNA molecules (median size > 250 kb) (Levy-Sakin and Ebenstein, 2013). It is a preamplification-free high-resolution technique and has been recognized as a key genetic technology for the detection of all classes of SVs such as aneuploidy, deletion, duplication, inversion, translocation, insertion, and complex rearrangement. OGM has recently been employed in the study of genetic diseases, hematological and solid tumors, and especially in the field of reproductive genetics (Mantere et al., 2021; Neveling et al., 2021; Sahajpal et al., 2021). In other studies, OGM has been utilized to unravel the relationship between gene and phenotype according to breakpoint locations (Chen et al., 2020; Wang et al., 2020; Lühmann et al., 2021; Mantere et al., 2021; Neveling et al., 2021)**.** In this study, we show that high-resolution OGM quickly and accurately detects chromosomal rearrangements in RSA patients. We reveal breakpoint regions at the gene level, providing new strategies for clinical genetic diagnosis.

## Materials and methods

### Subject recruitment

Seven couples with RSA were recruited. All aborted embryos were diagnosed as deletions or duplications based on CMA/CNV-seq results except case 02. Peripheral blood samples (5–10 mL) were collected for all laboratory tests after obtaining written informed consent. The experimental protocol was approved by the Ethics Committee of Jiangxi Maternal and Child Health Hospital (approval number EC-KT-202216).

### UHMW gDNA isolation and labeling

Ultra-high-molecular-weight (UHMW) genomic DNA (gDNA) was extracted from peripheral blood collected in EDTA anticoagulant tubes using the Bionano Prep SP Blood and Cell Culture DNA Isolation Kit (#80030; Bionano Genomics). UHMW gDNA was then quantified by the Qubit dsDNA assay BR kit with a Qubit 3.0 Fluorometer (Thermo Fisher Scientific), which is designed for a DNA concentration between 36 and 150 ng/μL. Pulsed-field gel electrophoresis was used to validate the integrity and size of the isolated gDNA. A total of 1 μg UHMW DNA was labeled using DLS DNA Labeling Kit (#80005; Bionano Genomics). 750 ng of gDNA was labeled by Direct label enzyme (DLE-1) and DL-green fluorophores. The labeled DNA was quantified to a recommended DNA concentration of 4–12 ng/μL before loading into the flow cell of the Saphyr chip (Bionano Genomics).

### Data collection, assembly and SVs calling

Raw DNA molecules were filtered, and only those with a molecular length greater than 150 kb and a minimum label density of nine labels per 100 kb were kept. The assembly algorithms aligned molecules *de novo* to construct a consensus genome map. The unique optical genome map was aligned to the human reference genome (GRCh38/hg38). SVs calling was performed using Bionano Solve v3.5.1 (Bionano Genomics). Data analysis was carried out by Access 1.7 Standalone software based on the Saphyr system (Bionano Genomics). The minimal breakpoint region was defined by the boundary of the DLE marker position closest to the crossover point on each chromosome. The bnx file generated after each run of each flowcell was used to generate the molecule quality report meeting the following parameters: N50 > 230 kbp; effective coverage depth >×80; average label density between 14 and 17; map rate ≥70%; positive label variance between 3% and 10%; and negative label variance between 6% and 15%.

### Oxford Nanopore sequencing

Phenol chloroform extraction was used to extract gDNA from peripheral blood samples. DNA was precipitated with isopropyl alcohol and washed with ethanol. The mixture was then purified using AMpure XP beads (#A63882, Beckman Coulter). The DNA was quantified with a Nanodrop spectrophotometer and Qubit 3.0 Fluorometer (#Q33216, Life Invitrogen) and assessed for quality and integrity using gel electrophoresis. Then, the DNA was end-repaired and A-tailed according to the Oxford Nanopore Technology (ONT) instructions. The purified DNA library was loaded onto an ONT sequencing flow cell (FLO-PRO002) and run on an ONT sequencer (PromethION 48). Basecalling was performed using ONT’s Guppy v6.2.1 Guppy basecaller software, and the resulting reads were filtered for quality using ONT’s Albacore software. The high-quality reads were then aligned to the human reference genome (GRCh38/hg38) using minimap2 alignment software. The final results were visualized using Integrative Genomics Viewer (IGV).

### G-banding karyotyping

Heparin tubes were used to collect 0.3–0.5 mL of peripheral blood. Tubes were inoculated into 1,640 culture medium and incubated at 37°C for 72 h. Colchicine was added 4 h before harvest to yield a final concentration of 0.08 μg/mL. Standard cytogenetic techniques were applied to prepare the cells in metaphase. The Leica GSL-120 automatic chromosome scanning system was used to count at least 20 cells and analyze 5 karyotypes. In cells with mosaicism, the number of counting cells were increased to 50. The chromosome karyotypes were identified according to the International System for Human Cytogenetic Nomenclature (ISCN) 2020.

### FISH

Cells in metaphase were fixed on clean slides according to the G-banding procedure (acetic acid:ethanol = 1:2), baked at 52°C for 2 h, and dehydrated in an ethanol concentration gradient. The hybridization reaction system was prepared as directed by the manufacturer (Abbott Molecular, United States). To make the total volume of the hybridization buffer 10, 1 μL probes were added. Samples were denatured with the chromosome specimens on slides at 72°C for 5 min in an incubator and then hybridized in a black wet box at 42°C for 12 h. The slides were then washed for 2 min with 0.4 × SSC/0.3% NP-40 at 73°C, 2 × SSC/0.1% NP-40 for 2 min at room temperature, then allowed to air dry. To the hybrid region, 10 μL DAPI were added, and fluorescent signals were analyzed using a fluorescence microscope.

### CMA analysis

Abortion tissues were collected to extract gDNA for CMA analysis. A CytoScan 750K chip (Affymetrix, United States) was used to detect the DNA samples of villi. Villi samples contaminated with more than 30% maternal cells were excluded. In brief, DNA was digested, ligated, amplified using PCR, purified, quantified, fragmented, labeled, hybridized, washed, stained, and scanned according to the manufacturer’s instructions. Data were processed and analyzed using the GenomeStudio software (Illumina). CNVs pathogenicity was labeled according to the American College of Medical Genetics and Genomics (ACMG) and Clinical Genome Resources Institute (ClinGen) guidelines. CNVs results were divided into the following categories: pathogenic (P); likely pathogenic (LP); variant of uncertain significance (VOUS); likely benign (LB); and benign (B).

## Results

### Patient demographics

**FIGURE 1:**
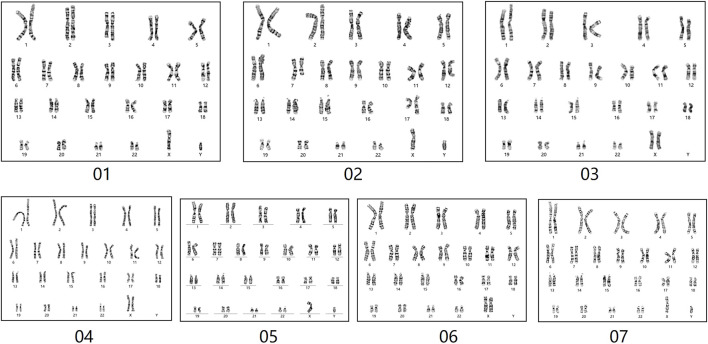
The karyograms of seven patients.

One partner in each of the seven couples had experienced two spontaneous abortions, and of these, three patients had abnormal karyotypes and four couples had normal karyotypes. Except for one male who was reexamined for chromosomal abnormality, the couples' abortion tissues were tested for copy number variations (Table 1; Figure 1).

**TABLE 1:** Patient demographics, karyotypes, and CNVs of miscarriage tissues.

Patient	Gender	Karyotype	Patient age in years	Partner age in years	Miscarriages	CNVs of miscarriage tissue (GRCh38/hg38)
01	Male	46,XY,t(3;8)?(q28;p22)	33	30	2	6q25.1(149,547,655-150,890,234) × 1 (VOUS)^a^ 8p23.3p22(1,710,455-13,638,023) ×3 (P)
02	Male	46,XY,inv(7)(q31.3q32)t(7;17)(q31.3;q25)	30	29	3	—
03	Female	46,XX,t(10;16)(p13;q24)	38	40	2	10q26.12q26.3(120,725,578-133,612,882) × 1 (P) 16q23.2q24.3(80,632,478-90,088,654) × 3 (P)
04	Female	46,XX	31	32	2	13q14.2q34(49883309-114344353) × 1 (P) 14q21.2q32.33(45360061-106851686) × 3 (P)
05	Male	46,XY	31	31	2	6q25.3q27(159,326,441-170,605,209) × 3 (LP)
06	Female	46,XX	32	32	3	1q42.2-q44(231,267,102-249,240,147) × 3 (P) 11q23.3-q25(119,548,730-134,945,944) × 1 (P)
07	Male	46,XY	33	31	2	1p36.33p36.32(914087-2544379) × 3 (LP) 9q34.3(137002730-138124196) × 1 (P)

^a^ The deletion inherited from the partner.

### Optical genome mapping analysis of SVs

The QC parameters of seven samples are summarized in Supplementary Table S1. OGM generated an average of 1563.0 Gb (range 1152.0–1946.8 Gb) of data per sample. The average N50 molecule length (≥150 kb) was 286.6 kb (range 261.8–312.0 kb). The average mapping rate was 89.6% (range 78.1%–90.9%), and the average labeling rate was 15.0 labels (range 14.6–15.7) per 100 kb. The average effective coverage depth was 488.9× (range 329.8–563.3×). The SVs were called by comparing maps and identifying discrepancies. SV calling detected an average of 5,937 SVs (range 1,942–6,668) per sample, the vast majority of which were insertions and deletions (average 3,667 and 1,557, respectively) (Supplementary Figure S1).

**FIGURE 2:**
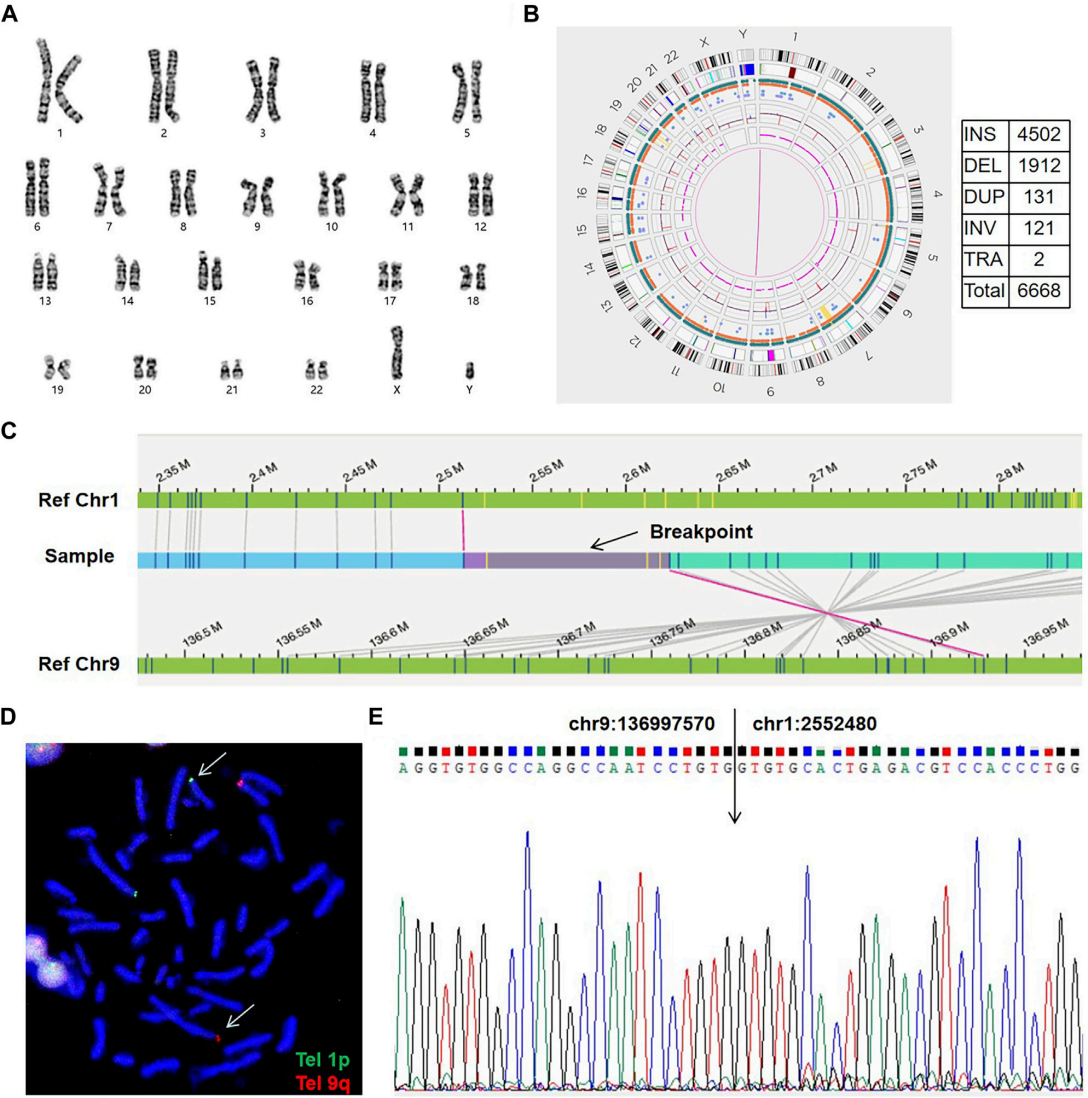
An example of a cryptic BRT (sample 07). **(A)** The karyogram shows a normal karyotype. **(B)** Circos plot and SVs. The pink line connecting chr1 and chr9 shows a translocation. SV, structural variation; INS, insertion; DEL, deletion; DUP, duplication; INV, inversion; TRA, translocation. **(C)** Genome map view indicates the translocation of t(1; 9)(p36.32; q34.3) and shows the breakpoint (black arrow). Aberrant molecules support the translocation. **(D)** FISH results using telomere probes 1p and 9q for the metaphase chromosome. Arrows show the translocation chromosomes.Tel, telomere probes. **(E)** Sanger sequencing shows the exact breakpoint location.

**FIGURE 3:**
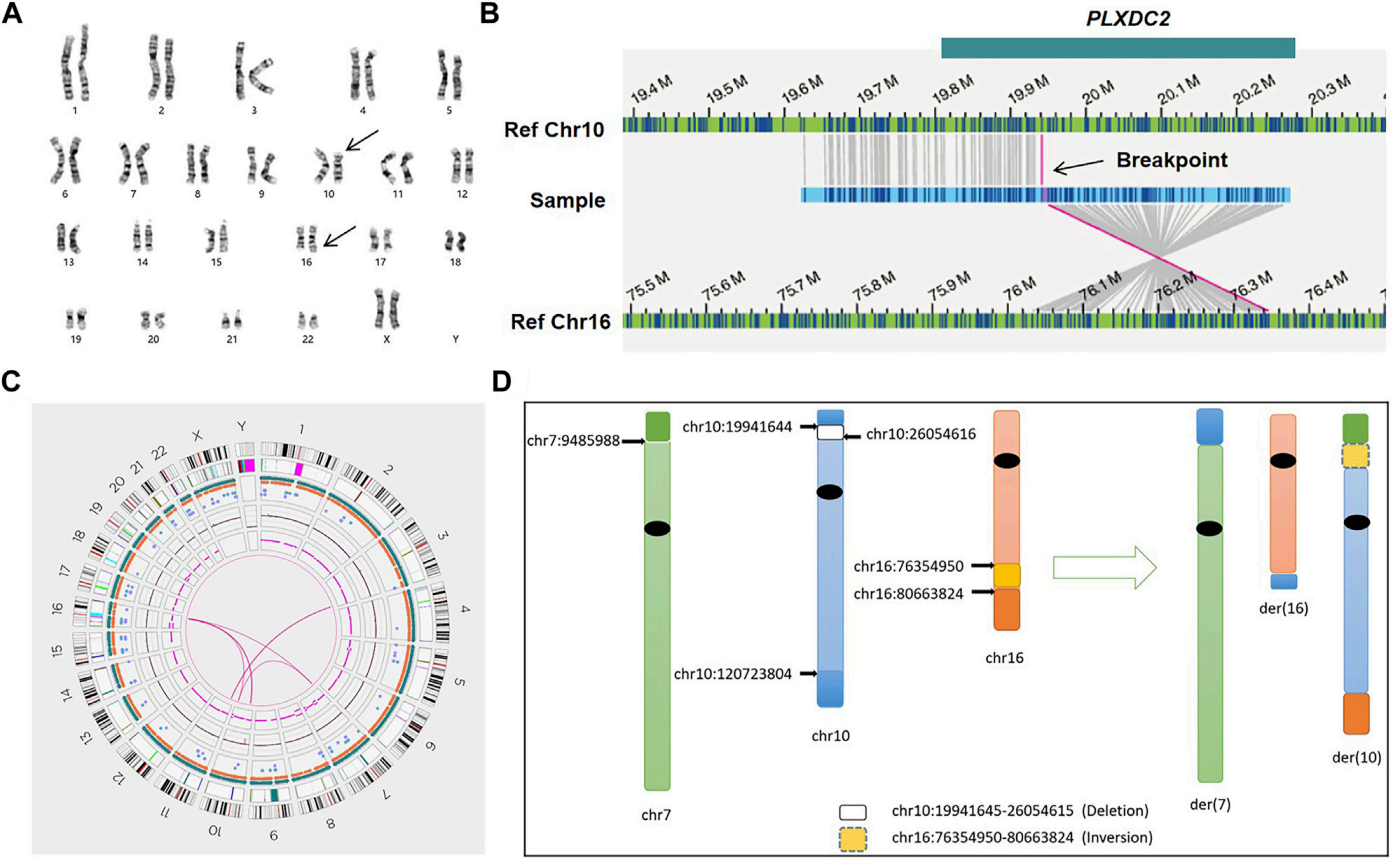
Genome-wide visualization of OGM data and an example of CCR (sample 03). **(A)** Karyogram. Arrows show the translocation t(10; 16)(p13; q24) at the cytogenetic level. **(B)** The genome map view shows one of the translocations t(10; 16)(p12.31; q23.1) and the respective disrupted gene (GRCh38/hg38). **(C)** Circos plot. The pink line connecting chr3 and chr11 shows a balanced translocation, and the pink lines among chr7, chr10, and chr16 indicate a complex chromosomal rearrangement. **(D)** Idiograms and exact breakpoints for the CCR among chr7, chr10 and chr16.

For all patients, we successfully detected the respective SVs with OGM. The average minimum coordinates of breakpoints regions identified was 18.6 kb (range 2.6–44.6 kb). There were four samples with normal karyotypes identified as BRT by OGM, including three cryptic BRTs (Patient 05, 06 and 07) and one BRT missed by karyotyping due to similar banding patterns (Patient 04). An example of a cryptic BRT [t(1; 9)(p36.32; q34.3)] identified by OGM is shown in Figure 2. One case previously identified as BRT was detected as insertional translocation by OGM (Patient 01). Karyotyping revealed that there was both inversion and BRT in case 02; however, OGM detected this as a simple BRT. Unexpectedly, karyotyping identified case 03 as a simple BRT [46,XX,t(10; 16)(p13; q24)], but OGM identified them as both a cryptic BRT and CCRs among five chromosomes (Figure 3). Within these breakpoint regions, we identified twelve potentially interrupted genes, two of which are related to male infertility and embryo transport (Camprubí et al., 2016; Bianchi et al., 2021), namely, *FOXK2* in the 17q regions of patient 02 and *PLXDC2* in the 10p regions of patient 03 (Table 2).

**TABLE 2:** Detailed characterizations of the structural variations and breakpoints in our study.

Patient	Karyotype	Molecular karyotype	OGM		ONT + Sanger sequencing^b^	
			Minimum coordinates of BP regions (size in Kb)^a^	Gene mapping in BP regions	BP coordinate	Gene disrupted (position)
01	46,XY,t(3;8)?(q28;p22)	ogm[GRch38]ins(3;8)(q26.33;p23.3p22)	chr3:179300066–179315579 (15.5)	—	chr3:179306381	—
			chr8:1725258–1735725 (10.5)	—	chr8:1733616	—
			chr8:13631460–13646784 (15.3)	—	chr8:13644413	—
02	46,XY,inv(7)(q31.3q32)t(7;17)(q31.3;q25)	ogm[GRch38]t(7;17)(q31.32;q25.3)	chr7:122569155–122591342 (22.2)	*CADPS2*	chr7:122570692	*CADPS2*
			chr17:82547659–82550216 (2.6)	*FOXK2*	chr17:82549192	*FOXK2*
03	46,XX,t(10;16)(p13;q24)	ogm[GRch38]t(3;11)(q27.1;p14.3),der(7)t(7;16;10)(p21.3;q23.2;q26.12),der(10)del(10)(p12.31p12.1)t(10;16)(p12.31;q23.1)t(7;16;10),der(16)t(10;16)t(7;16;10)	chr3:183886373–183923324 (37.0)	*ABCC5*	chr3:183912450	*ABCC5*
			chr11:22905493–22934527 (29.0)	—	chr11:22908819	*—*
			chr7:9482138–9494105 (12.0)	—	chr7:9485988	*—*
			chr10:120715571–120730013 (14.4)	—	chr10:120723804	*—*
			chr10:26036003–26058923 (22.9)	*MYO3A*	chr10:26054616	*MYO3A*
			chr10:19941594–19958974 (17.4)	*PLXDC2*	chr10:19941644	*PLXDC2*
			chr16:76346136–76368415 (22.3)	*CNTNAP4*	chr16:76354950	*CNTNAP4*
			chr16:80622483–80667034 (44.6)	*CDYL2*	chr16:80663824	*CDYL2*
04	46,XX	ogm[GRch38]t(13;14)(q14.2;q21.2)	chr13:49891734–49896984 (5.3)	*CTAGE10P*	chr13:49892537	*CTAGE10P*
			chr14:45351567–45372726 (21.2)	*—*	chr14:45366646	—
05	46,XY	ogm[GRch38]t(2;6)(q37.3;q25.3)	chr2:242145302–242151438 (6.1)	*LINC01881*	chr2:242146666	*LINC01881*
			chr6:159306493–159316621 (10.1)	*—*	chr6:159315166	*—*
06	46,XX	ogm[GRch38]t(1;11)(q42.2;q23.3)	chr1:231166679–231210794 (44.1)	*TRIM67*	chr1:231203504	*TRIM67*
			chr11:119693143–119707121 (14.0)	*NECTIN1*	chr11:119711245	*NECTIN1*
07	46,XY	ogm[GRch38]t(1;9)(p36.32;q34.3)	chr1:2512657–2524460 (11.8)	*PANK4*	chr1:2552480	*—*
			chr9:136928032–136940005 (12.0)	*—*	chr9:136997570	*—*

^a^ The minimum breakpoint was defined as the distance between the opposing CTTAAG, label sites on either side of the breakpoint of the two chromosomes involved in the translocation.

^b^ The breakpoints of cases 01–06 were validated by ONT, and case 07 was by Sanger sequencing.

### Validation of breakpoint locations by Oxford Nanopore sequencing

**FIGURE 4:**
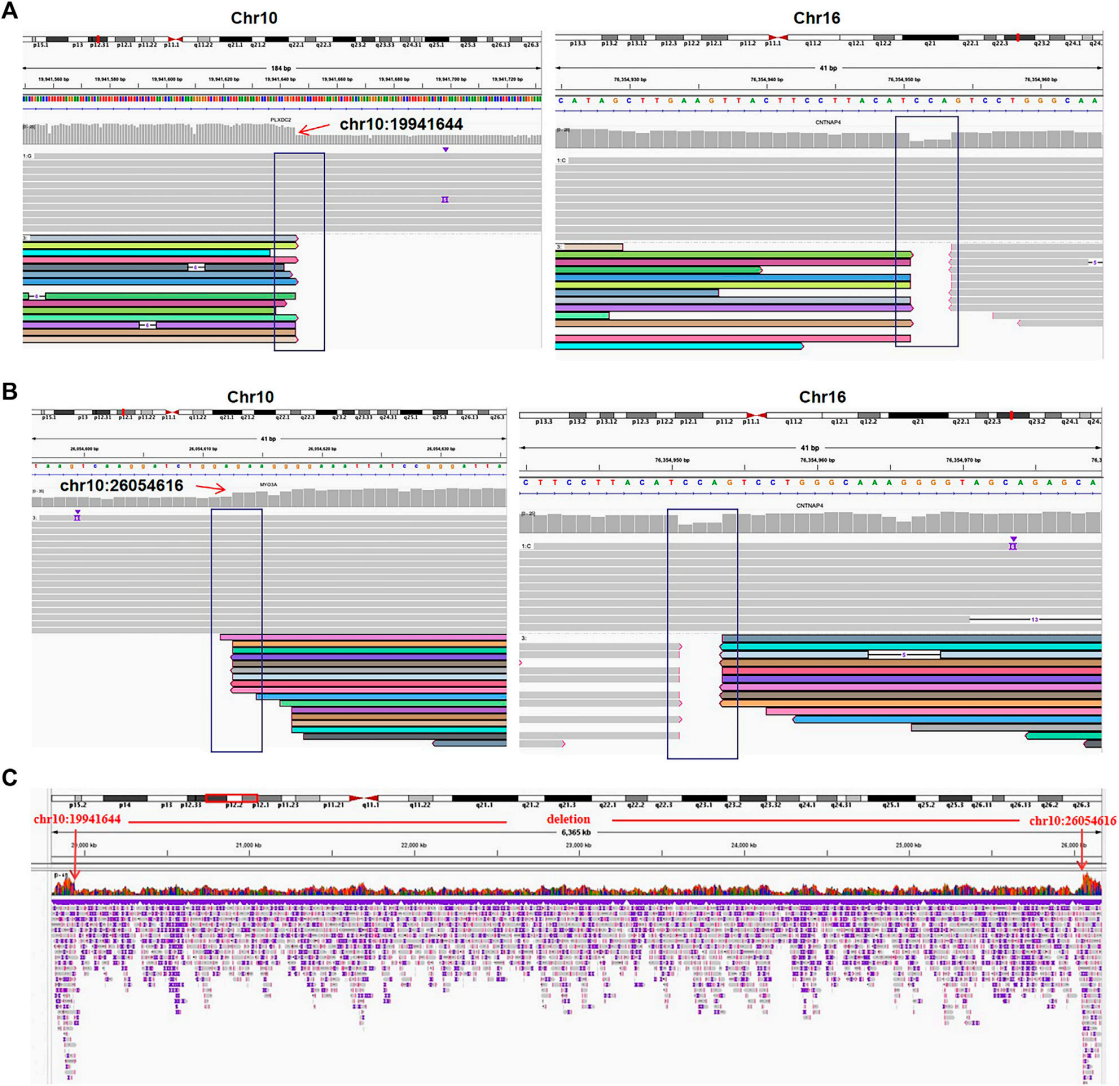
The translocation and deletion confirmed by Oxford Nanopore sequencing for case 03. **(A)** Read mapping of translocation breakpoints [t(10; 16)(p12.31; q23.1)]. DNA fragments were compared to human genome reference GRCh38/hg38, and the breakpoints were shown in integrative genomics viewer (IGV). **(B)** IGV for the translocation [t(10; 16)(p12.1; q23.1)]. **(C)** IGV of chr10 shows a deletion of 10q12.31q12.1 (chr10:19941644-26054616).

Long-read sequencing provided by ONT was used to confirm the OGM results. All breakpoints were accurately located to a resolution of single nucleotide (Table 2; Figure 4). All nucleotide sequence locations were successfully mapped to the fractured chromosomes, and all the breakpoint coordinates detected by ONT were within the breakpoint regions provided by OGM except in case 07, which was validated by Sanger sequencing. The disrupted genes predicted by the OGM within the breakage regions were consistent with those detected by ONT, except in case 07. The ONT QC parameters are summarized in Supplementary Table S2.

## Discussion

RSA affects millions of couples worldwide, placing a financial and psychological burden on affected couples. Chromosomal abnormalities are recognized as the main genetic cause of RSA, accounting for 60% of all cases (Levy et al., 2014). Karyotyping is a basic routine diagnostic for RSA samples and is indicated for detecting large fragments of SVs as well as numerical aberrations. However, its overall diagnostic rate is well below 10% (De Braekeleer and Dao, 1990; 1991; Chantot-Bastaraud et al., 2008; Hofherr et al., 2011). Many cryptic chromosomal abnormalities are undetectable due to poor resolution and variations in sample preparation and laboratory quality. Although karyotyping may be combined with chromosomal microarray analysis to improve diagnosis rates, this approach does not improve the resolution of the detection of balanced SVs, making it difficult to fully reveal the genetic cause of RSA. The advent of the OGM resolved this problem. As a new generation of genomic analysis technology, OGM detects all classes of SVs, as well as CNVs (Sahajpal et al., 2021). Additionally, it can accurately detect breakpoint regions within 10 kb (Wang et al., 2020). In this study, we have successfully detected all SVs using OGM, including one case with both cryptic BRT and CCRs. OGM identified RSA-related chromosomal abnormalities with a higher diagnostic rate and resolution than conventional genetic investigation tools.

BRTs are common chromosomal abnormalities, occurring in about 2.2% patients who experienced RSA. The accurate determination of breakpoints is very important for assisted reproductive technology (Jacobs et al., 1992). Cryptic BRTs are SVs that do not obviously change chromosome banding or the translocation segments are below the karyotyping limit. Indeed, they are undetected by standard-of-care tests and may be underestimated. A recent study has shown that OGM detects cryptic BRTs quickly, accurately, and with high resolution (Zhang et al., 2023). In this study, we successfully detected four cryptic BRTs with fragment sizes ranging from 1.4 to 22.9 Mb. All breakpoints were confirmed by sequencing. Currently, all patients are undergoing preimplantation genetic testing (PGT) process.

In addition to cryptic BRTs, a large proportion of apparently balanced translocation include additional complexity that cannot be detected by routine clinical methods and are therefore often misdiagnosed (Wilch and Morton, 2018). In case 01, the patient’s partner experienced two spontaneous abortions. The karyotype of the couple were 46, XX and 46,XY,t(3; 8)?(q28; p22) respectively, but the breakpoints were uncertain. However, CNVs of the aborted embryo showed a duplication of 8p23.3p22 and a deletion of 6q25.1. OGM was used to refine the karyotype, which detected three breakpoints and a deletion (Chr6:149536442 -150889382) in the male and female respectively. These results were confirmed by Oxford Nanopore sequencing. The circos plot displays an insertion between chromosome 8 and chromosome 3 (Supplementary Figure S2). Insertion is a type of chromosomal translocation that typically involves three breakpoints. In contrast to the common translocations of chromosome ends, these translocation segments are inserted into the breakpoint regions of a nonhomologous chromosome (Weckselblatt and Rudd, 2015), generating four kinds of gametes during meiosis: two with the normal amount of genetic material, one with partial trisomy, and one with partial monosomy. This can lead to RSA or disruption of offspring after development into embryos. The OGM elucidates complex cases that were undetectable by karyotyping and helped RSA patients to select better reproduction patterns.

Other rare chromosomal abnormalities in clinic are CCRs, which are structural variants arising from at least three breakpoints and are often not adequately characterized by conventional G-band karyotyping or other clinical molecular analyses. They can be classified as balanced or unbalanced according to whether there is chromosome material loss or gain (Sinkar and Devi, 2020; Yang and Hao, 2022). Carriers of CCRs are at high risk of infertility, sub-fertility, or recurrent spontaneous abortions (Xing et al., 2022). They also have high probability of developmental delay if the CCRs are unbalanced or some pathogenic genes are interrupted by position effects (Ngim et al., 2011; Harton and Tempest, 2012). In this study, we detected a CCR in case 03. The patient was a 38-year-old female who underwent three spontaneous abortions. Karyotyping of the patient revealed apparently balanced translocation involving 10p12 and 16q23, while the CMA result of the abortion tissue was: 10q26.12q26.3 (120,725,578–133,612,882) × 1; 16q23.2q24.3 (80,632,478–90,088,654) × 3. The translocation breakpoints by revealing a complex rearrangement among chr7, chr10 and chr16, and a cryptic translocation between chr3 and chr11. In addition, OGM detected deletion of 10p12.31p12.1, so we redefined the patient as an unbalanced CCR carrier. Our findings showed high consistency between the OGM and the sequencing results and further refined the complex karyotypes. Because of the unique recurrence risk of CCRs, we recommend that the patient use donor eggs to produce offspring for her future reproductive decisions.

Genes disrupted by translocation breakpoints may constitute candidate genes for diseases such as male infertility, intellectual disability, and other congenital abnormalities (Aristidou et al., 2017; Wang et al., 2020). In this study, we found disruptions in sequences of *CADPS2*, *FOXK2*, *ABCC5*, *MYO3A*, *PLXDC2*, *CNTNAP4*, *CDYL2*, *CTAGE10P*, *LINC01881*, *TRIM67*, *NECTIN1* and *PANK4* across the six samples. All disrupted genes were consistent with those detected by ONT, except *PANK4* found in case 07. This inconsistency may be due to the methyltransferase DLE-1 producing a relatively low number of labels in the human genome (approximately 14–15 markers per 100 kb) (Dremsek et al., 2021), as determining the coordinates of breakpoints regions is difficult when there are no labels around those areas. In case 02, the breakpoint in 17q25.3 interrupted the *FOXK2* gene*,* which is a transcription factor and has been found to have decreased methylation in infertile men (Camprubí et al., 2016). In case 03, the breakpoint in 10p12.31 interrupted the *PLXDC2* gene, which is an activation ligand for the G-protein coupled receptor Adgrd1 displayed on cumulus cells. In a mouse model, *PLXDC2* was shown to play a role in controlling embryo transit through regulating oviductal fluid flow together with Adgrd1 (Bianchi et al., 2021). These findings shed new light on the relationship between certain genes and RSA.

In summary, OGM is a complement to conventional methods, especially in the detection of cryptic BRTs and CCRs. The clinical application of OGM allows for a more robust analysis of genetic abnormalities in RSA patients, thereby improving the diagnosis rate. In addition, more information about the breakpoint regions can be provided at the gene level, which can be used to help elucidate the relationship between location effects and disease in clinical work.

## Data Availability

The data presented in the study are deposited in Figshare https://doi.org/10.6084/m9.figshare.24046965.
