# Supplementary material for: Analysis of chromosomal structural variations in patients with recurrent spontaneous abortion using optical genome mapping
Source: Front Genet. 2023 Sep 4;14:1248755. doi: 10.3389/fgene.2023.1248755 (PMC10507169; doi:10.3389/fgene.2023.1248755)
Supplement: Supplementary file 1 [file Table4.DOC]

Supplementary Table 2 The ONT QC parameters of all samples

| Sample | Pass bases | Mapped Reads | Depth (X) | Mean length (bp) | N50 (bp) |
| --- | --- | --- | --- | --- | --- |
| 01 | 90,874,618,673 | 5,672,723 | 31.7 | 16,020 | 28,348 |
| 02 | 103,703,605,914 | 4,699,630 | 36.3 | 20,103 | 29,973 |
| 03 | 67,765,463,251 | 2,607,023 | 22.6 | 25,929 | 37,758 |
| 04 | 104,159,345,302 | 6,900,014 | 36.4 | 15,096 | 26,972 |
| 05 | 108,270,149,460 | 4,567,620 | 37.8 | 23,704 | 35,066 |
| 06 | 102,794,818,193 | 5,018,864 | 36.0 | 20,482 | 32,866 |
