# Supplementary material for: Analysis of chromosomal structural variations in patients with recurrent spontaneous abortion using optical genome mapping
Source: Front Genet. 2023 Sep 4;14:1248755. doi: 10.3389/fgene.2023.1248755 (PMC10507169; doi:10.3389/fgene.2023.1248755)
Supplement: Supplementary file 2 [file Table3.DOC]

Supplementary Table 1 The Bionano QC parameters of all samples

| Sample | Total length (Gb) | Molecular N50 (kb) | Label density (/100kb) | Effective coverage  (X) | Map rate (%) |
| --- | --- | --- | --- | --- | --- |
| 01 | 1154.0 | 312.0 | 14.9 | 329.8 | 88.2 |
| 02 | 1152.0 | 287.3 | 15.7 | 339.2 | 90.9 |
| 03 | 1186.1 | 287.3 | 15.0 | 343.2 | 89.7 |
| 04 | 1184.2 | 278.3 | 15.5 | 342.0 | 89.2 |
| 05 | 1946.8 | 303.0 | 14.6 | 563.3 | 90.2 |
| 06 | 1934.8 | 277.1 | 14.8 | 560.1 | 89.4 |
| 07 | 1926.9 | 261.8 | 14.6 | 487.3 | 78.1 |
