# Supplementary material for: Analysis of chromosomal structural variations in patients with recurrent spontaneous abortion using optical genome mapping
Source: Front Genet. 2023 Sep 4;14:1248755. doi: 10.3389/fgene.2023.1248755 (PMC10507169; doi:10.3389/fgene.2023.1248755)
Supplement: Supplementary file 3 [file Table1.DOC]

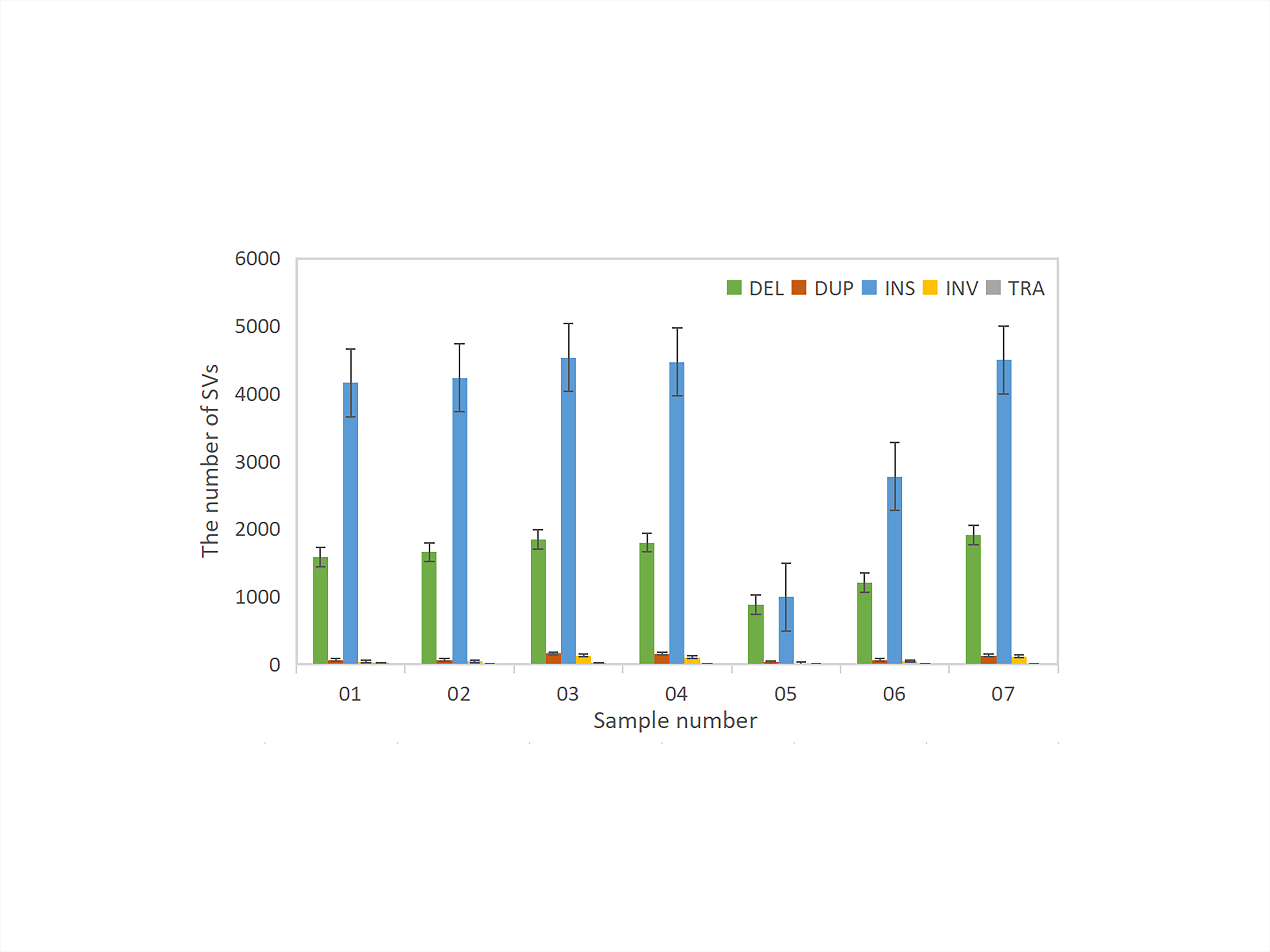


**Supplementary Figure S1** The type of SVs identified in all samples. SV, structural variation; DEL, deletion; DUP, duplication; INS, insertion; INV, inversion; TRA, translocation
