# Supplementary material for: Analysis of chromosomal structural variations in patients with recurrent spontaneous abortion using optical genome mapping
Source: Front Genet. 2023 Sep 4;14:1248755. doi: 10.3389/fgene.2023.1248755 (PMC10507169; doi:10.3389/fgene.2023.1248755)
Supplement: Supplementary file 4 [file Table2.DOC]

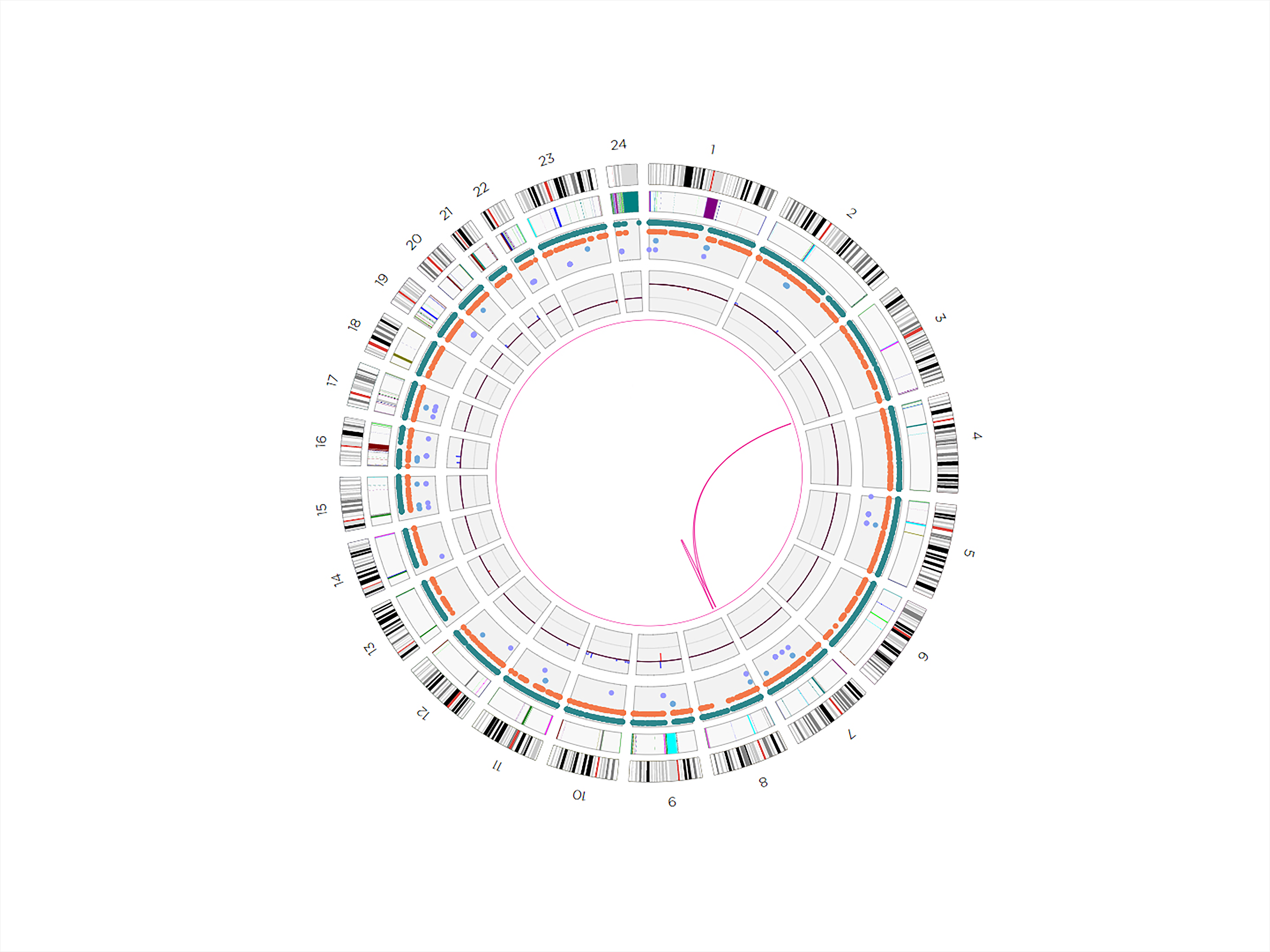


**Supplementary Figure S2** Circos plot and de novo SVs after filtering-out in sample 01.The pink line shows an insertion between chr3 and chr8.
